# Un néo de la vulve chez une patiente VIH séropositive: à propos d'un cas historique

**DOI:** 10.11604/pamj.2014.18.33.3814

**Published:** 2014-05-08

**Authors:** Sarah Amourak, Fatimazehra Fdili Alaoui

**Affiliations:** 1 Service de Gynécologie Obstétrique II, CHU Hassan II, FES, Maroc

**Keywords:** néoplasme, VIH, vulve, Neoplasm, HIV, vulva

## Image en medicine

C'est une patiente âgée de 28 ans, suivie depuis 3 ans pour infection à VIH initialement stade C2 mise sous tri thérapie qui présente depuis 3mois des lésions génitales. L'examen trouve une importante masse bourgeonnante, saignant au contact occupant toute la surface de la vulve, la région périnéale et le pourtour de l'anus, avec une adénopathie inguinale. La TDM TAP: pas de localisations secondaires. La biopsie de la tumeur: carcinome épidermoide bien differencié infiltrant associé à des lésions de carcinome in situ en surface. Le cancer de la vulve survient le plus souvent après l’âge de 70 ans, mais des femmes beaucoup plus jeunes peuvent également être atteintes. Il peut se manifester par une irritation chronique de la vulve, démangeaisons, sensation de brûlure au niveau des lèvres génitales, dyspareunie ou encore présence de zone décolorées sur les lèvres. Il n'existe pas de test de dépistage, mais l’évaluation attentive de la vulve lors de l'examen gynécologique permettrait de découvrir la plupart des cancers et lésions précancéreuses de la vulve. L'infection par le VIH favorise la persistance des virus HPV et ainsi l'apparition du cancer de la vulve, on note une trentaine de cas de cancers de la vulve+ VIH qui ont été rapportés ce jour, ces images illustrent un cas historique d'un cancer de la vulve.

**Figure 1:**
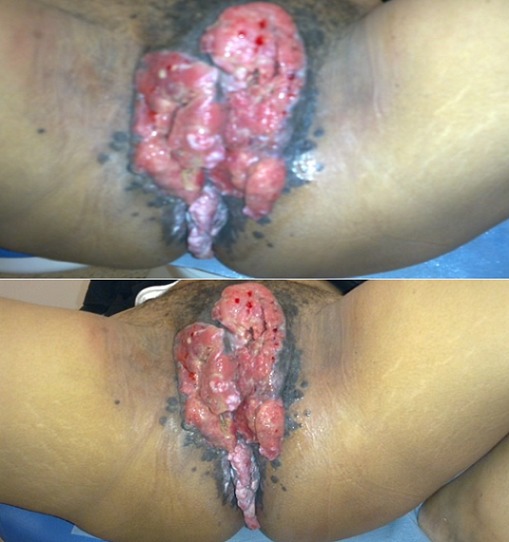
Masse bourgeonnante,occupant toute la surface de la vulve, la région périnéale et le pourtour de l'anus

